# Effect of Laser Heating on the Life of Cutting Tools Coated with Single- and Multilayer Coatings Containing a TiN Layer

**DOI:** 10.3390/ma15114022

**Published:** 2022-06-06

**Authors:** Maciej Jan Kupczyk, Jerzy Józwik

**Affiliations:** 1 Institute of Mechanical Technology, Poznan University of Technology, 60-965 Poznan, Poland; maciej.kupczyk@put.poznan.pl; 2 Faculty of Mechanical Engineering, Lublin University of Technology, 20-618 Lublin, Poland

**Keywords:** hard coatings, tool life, adhesion, laser heating, cutting tools

## Abstract

This study proposes a novel use of laser heating to increase the adhesion between coatings fabricated by low-temperature PVD and replaceable cemented carbide cutting inserts, thus extending the life of these cutting tools in the machining of difficult-to-machine materials. Our previous studies conducted on CVD coatings showed that these coatings had higher adhesion due to a much higher process temperature. However, taking into account the fact that PVD coatings have better technological properties (e.g., lower structure porosity, higher hardness, and better tribological properties) than CVD coatings, it is fully justified to investigate ways of improving the PVD coating adhesion to the substrate. In this study, replaceable cutting inserts with different hard coatings of titanium nitride were used. Laser heating was conducted with different power densities. The adhesion strength of the tested coatings was determined via vibration spectrum analysis. In addition, 2D surface imaging, scanning electron microscopy, and X-ray fluorescence spectrometry were employed to examine the coatings after laser heating. A significant increase in the adhesion of single-layer (TiN) and double-layer (TiCN + TiN) coatings to the cemented carbide substrate, together with increased tool life, was observed after heating the samples with 40% of the maximum laser power. The application of a multilayer coating containing thermal shock-sensitive (TiAlSi) N did not increase the tool life. This paper attempts to interpret the obtained results.

## 1. Introduction

The selection of a coating material in terms of its chemical composition, hardness, high melting point, brittle fracture resistance, proper coating structure, and friction coefficient is not enough for the coating deposited on a cutting blade to effectively perform its anti-wear function. In addition to the above, it is also necessary to ensure that the coating has a sufficiently high adhesion to the substrate [1,2,3,4,5,6,7,8,9]. The coating with required mechanical properties becomes useless if its adhesion to the substrate is insufficient. Due to very high unit pressures occurring in the cutting zone, coated cutting blades must meet special requirements regarding coating–substrate adhesion. If the coating adhesion to the substrate is not sufficient, the coating is almost immediately removed from the cutting tool blade where the workpiece and chip interact [10,11,12].

Currently, physical vapor deposition (PVD) methods are widely used in the production of hard anti-wear coatings on cutting tools. This results from the fact that coatings produced using this technique have better technological and operational properties than CVD coatings. PVD coatings are characterized by, among other things, lower porosity, a more compact structure (higher hardness [13]), and a smoother surface (lower friction coefficient) [14].

Many factors have an impact on the adhesion strength of hard PVD coatings to the substrate (cutting blade). First of all, they include the types of coating and substrate materials, substrate surface morphology, coating process conditions (kinetic energy of the coating ions), type of substrate surface preparation (e.g., chemical etching, degreasing, low-temperature or high-temperature spraying), substrate temperature, difference between the values of the thermal expansion coefficient of the coating material and the substrate, and type of transition layer formed between the coating and the substrate (mechanical, discrete—monolayer on a monolayer, epitaxial, diffusion, compound, pseudo- diffusion, complex) [15].

As numerous studies have shown, in addition to the kinetic energy of the coating ions and the surface morphology, the temperature of the substrate is of particular importance for creating an appropriate transition layer in PVD processes to ensure high adhesion of the coating to the substrate. A sufficiently high temperature (selected in such a way that it does not lead to tempering of the base material) enables the creation of a favorable—diffusion or adhesive diffusion—transition layer. It should be emphasized that, with an increase in temperature, the tendency to form so-called diffusion adhesion increases [16]. The area of diffusion adhesion is characterized by gradual changes in the chemical composition, stresses, and constant lattice. The diffusion transition layer has desirable properties as a transition layer between very different materials, as it contributes to, inter alia, reducing mechanical stresses resulting from the disproportion of the thermal expansion coefficients of the coating materials and the substrate. An additional advantage of diffusion processes is that the gaps in the transition layer formed during the coating process can be filled with the material that covers the substrate [15].

Methods that may contribute to the formation of a diffusion or adhesive diffusion transition layer include heating the coating–substrate system using resistive heating, heating with high-frequency currents, arc heating, heating with capacitive current (dielectric heating), induction heating (heating with eddy currents), and laser heating [17,18,19,20,21,22]. Taking into account the above considerations, laser heating was used to increase the adhesion of selected coatings containing titanium nitride.

The use of laser-emitted radiation is now more and more widespread in various areas of production. Despite the fact that the laser was invented almost 70 years ago, the rates of progress in the research on laser technology and the design and manufacture of laser devices are constantly increasing. The growing interest in laser-emitted radiation is due to its specific, extremely useful properties and the possibility of constructing radiation sources with different parameters (wavelength, emitted power, continuous or pulsed mode of impact on the workpiece, etc.) [18,23].

There are two main advantages of using lasers in material processing [18,23,24,25,26,27,28]. The first advantage relates to the production of laser coatings (often known as plating or cladding in the case of laser-produced coatings) from powders, pastes, thin films, rods, and wires or by depositing sprayed suspensions [18,26,28,29,30]. The use of such coatings is primarily aimed at increasing the abrasive and erosive wear resistance, as well as heat and corrosion resistance, of materials [26,27,30,31,32,33]. These coatings are most often made on low-carbon and low-alloy structural steels [18,29,30].

The second advantage of using laser heating is the possibility of modifying the already produced coatings, i.e., for smoothing and sealing these coatings [26,30,31]. Smoothed coatings have increased resistance to both abrasive wear and scuffing [32,34]. For smoothing purposes, it is recommended to use laser beams with low-power densities and large diameters in order to melt layers to a small depth [23]. The laser beam can also be used to reduce the porosity of the coating by thickening the heated material and to eliminate scratches, delamination, or cracks on the coating surface [26,30]. Laser heating also reduces undesirable tensile stresses in the coating and substrate. With the tensile stresses reduced, the fatigue strength of the coating is increased [23].

This work is a continuation of previous studies on the effective use of laser heating to improve the adhesion of coatings to replaceable cemented carbide cutting inserts. Previous studies conducted by the coauthor of this paper [35,36] focused on coatings produced by the high-temperature CVD process (following the van Arkl and de Boer method improved by Münster and Ruppert) [37]. In contrast, PVD processes are low-temperature methods. Hence, for obvious reasons, the resulting coating adhesion to the substrate is often worse than that obtained from CVD processes. However, taking into account the advantages of using the PVD method in the production of anti-wear coatings (higher structure density, higher hardness, and better tribological properties than those of CVD coatings, etc.), it was considered necessary to improve the coating/substrate adhesion. Therefore, the novelty of this study in relation to previous studies and solutions is that it uses laser heating to effectively increase the adhesion of PVD coatings to replaceable carbide cutting inserts and—ultimately—to extend the service life of these inserts in turning processes for structural steel, a material which is widely used to produce machine parts requiring high mechanical strength and abrasion resistance.

As previously mentioned, the adhesion of the coating to the substrate is a key parameter affecting the life of coated cutting tools. In this paper, selected technological (adhesion and chemical composition) and operational (wear and tool life) properties of coated cutting tools were compared, before and after laser heating.

## 2. Materials and Methods

### 2.1. Cutting Inserts

Three types of coatings were deposited on the replaceable cutting inserts made of SM25 sintered carbide (P15–P40, M25–M35): TiN (single-layer coating), TiCN + TiN (double-layer coating), and TiN + (TiAlSi)N + TiN (triple-layer coating). The coatings were produced by the arc-plasma method, which is a PVD technique. The TiN coatings had an average thickness of 4.5 µm. The surface roughness before heating was Ra = 0.12 µm on average. The SE images of the coating fractures obtained at a magnification of 15,000× showed their compact structure, which is characteristic of area IV (T) according to the Thornton model.

**Figure 1:**
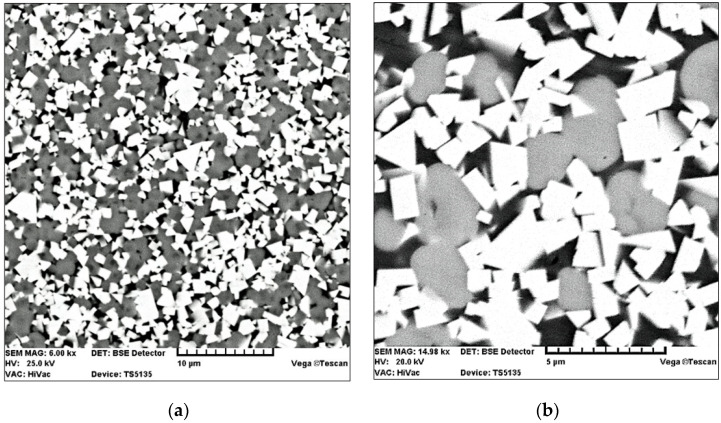
SEM images of the SM25 sintered carbide microsections at different magnification: (**a**) 6000×; (**b**) 15,000× [6,10].

SM25 sintered carbide has high bending strength. The sintered carbide cutting inserts were manufactured by BAILDONIT (Katowice, Poland). The material had an average grain diameter of 2 µm [6]. Table 1 gives data about the mechanical properties and chemical composition of SM25 cemented carbides [6]. Figure 1 shows SEM images of the microsection of the SM25 sintered carbide substrate.

### 2.2. Laser Heating Conditions

The coatings deposited on the sintered carbide substrate were laser-heated using a CO_2_ TLF 2600T laser test stand installed at the at the Laboratory of Laser Technology of the Institute of Mechanical Technology at Poznan University of Technology. This Trumpf’s CO_2_ molecular laser has a power of 2600 W. Its optical system of laser beam transmission is installed on a universal lathe, TUM35D1. The laser was equipped with the Laser Carl Zeiss LBK-FP2 laser head with a focal length of 87 mm.

Since the coatings had a similar color, the samples did not have to be coated with black gouache to maintain the same laser heating conditions. The TiN coating of all samples was light yellow. In previous studies [6,35], the samples were coated with black gouache before laser heating in order to ensure the same laser heating conditions. The laser heating process was conducted with a 4 mm diameter laser beam and a scanning speed of 1.3 m/min for the following laser power densities: (a) 4140, (b) 6210, (c) 8280, (d) 10,350, and (e) 12,420 W/cm^2^. The employed laser heating parameters are given in Table 2.

### 2.3. Measurements of Adhesion and Chemical Composition

**Figure 2:**
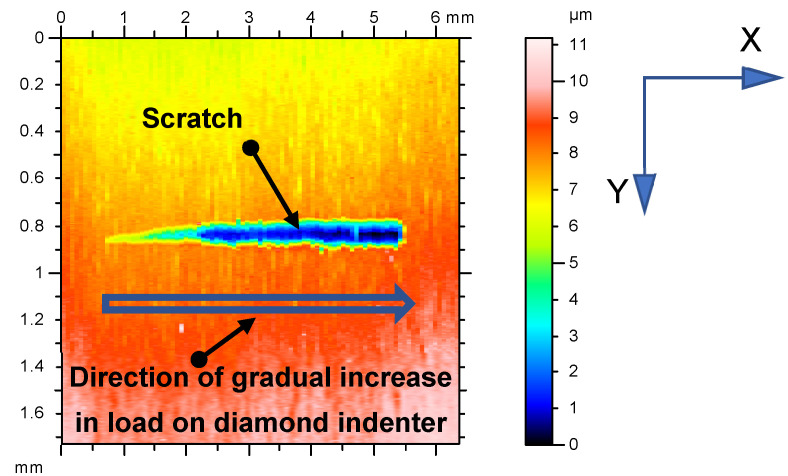
Image of scratch caused by gradual increase in load on diamond indenter (top view: load increase from left to right).

The adhesion of coatings before and after laser heating was measured using the UMT 2/3 Multi-Specimen Test System from Bruker Nano, Inc., Campbell, Arizona, USA, as well as our own designed test stand with two measuring channels [38]. The device is equipped with two channels for acoustic wave measurement by a sound level meter and vibration measurement by a piezoelectric transducer, and it is available in the Wear Resistant Coatings Laboratory at the Institute of Mechanical Engineering at Poznan University of Technology. The UMT 2/3 Multi-Specimen Test System is a multifunctional device which, on the basis of pressure, stylus displacement, lateral force, and acoustic emission, allows the user to automatically determine the coating adhesion to the substrate and measure the plastic and elastic deformation of the coating, as well as the coefficient of friction. It operates in three modes (scratch, pre-scan, and post-scan). The device allows computer processing of test results, their graphic visualization and archiving, and microscopic observation. The stand is equipped with a video system with a display unit and software. Using the above device, adhesion was evaluated via scratch test, which involved measuring the critical load *L_c_* at which the coating would peel off the substrate. Scratch testing is primarily used for evaluating the adhesion of hard and super-hard coatings to cutting blades [8,39,40,41,42,43,44,45]. To sum up, the critical load was estimated by measuring the actual value of *A_acc_* (the amplitude of the vibration acceleration signal), the vibration signal intensity, and its absolute value, as well as on the basis of images from the SEM and profilometer. Figure 2 shows an example image of a scratch mapped with a profilometer.

Scratch test parameters, proposed as a standard [6], were selected on the basis of the results of previous comprehensive studies investigating different types of coating materials and substrates for the cutting blade (1200 Rockwell diamond indenter). They were as follows: loading rate *dL*/*dt* = 300 N·min^−1^; scratching speed *dx*/*dt* = 7.5 mm·min^−1^; ratio dL/dx = 40 N·mm^−1^; diamond indenter radius *R* = 0.20 mm. These parameters ensured measurement repeatability and relatively high efficiency of the testing method. The values of adhesion (critical load *L_c_*) for the scratch test were determined as the mean of five measurements.

Adhesion results obtained from critical load measurements by the scratch test were verified by scanning electron microscopy (SEM) using a Tescan Vega 5135 (Brno—Kohoutovice, Czech Republic) and by measuring 2D and 3D profiles using the Waveline T8000 Hommelwerke profilometer produced by Hommelwerke GmbH (Alte Tuttlinger Straße 20, D-78056 VS, Schwenningen, Germany), as well as by energy-dispersive X-ray fluorescence (EDXRF) microanalysis conducted using an X-ray fluorescence spectrometer from the Helmut Fisher Institute Für Elektronik und Messtechnik (Industriestraße 21, 71069 Sindelfingen/near Stuttgart, Germany). With this instrument, elements were identified by X-ray fluorescence microanalysis in compliance with EN ISO 3497. This spectrometer was particularly suitable for the study due to the fact that it is a universal instrument for nondestructive, accurate, and easy measurements of chemical composition (X-ray fluorescence microanalysis) from a small area of thin and very thin coatings (<0.1 μm) made of hard materials deposited on cutting tools. This instrument can also be used for examining multilayer systems. In addition, it is used for assessing the chemical composition of volumetric materials and for measuring coating thickness. Moreover, the spectrometer allows computer processing of test results, their graphic representation and archiving, and microscopic observation.

### 2.4. Conditions of Wear and Tool Life Tests

This section presents selected results of the verification tests, considered to be most important for the problem under analysis. After determining their technological properties, the cutting inserts were subjected to performance tests. The SM25 sintered carbide cutting inserts coated with hard coatings were examined for their wear and life in a longitudinal turning process performed on the TK66 lathe.

After clamping the rectangular cutting inserts in the CSRNR 252509-ID tool holder, the following cutting insert geometry was obtained: cutting tool blade angle *K_r_* = 75°; clearance angle *α*_0_ = 6°; tool included angle *o_r_* = 90°; rake angle *γ*_0_ = −6°; cutting blade inclination angle *λ_s_* = −6°. Wear and tool life tests were conducted using the following machining conditions: cutting speed *v_c_* = 120 m/min; feed per revolution *f* = 0.2 mm/rev; depth of cut *a_p_* = 1 mm. To assess the wear of all tested cutting tools, the mean value of the wear index *VBc* from five tests (repetitions) was determined.

Due to their rectangular shape, the cutting inserts were easy to mount in the measuring instruments, which—in turn—made it possible to conduct a series of additional tests (validation). The next step of the study involved selecting the workpiece material. The 35HGS construction alloy steel for surface quenching and heat treatment (30–32 HRC hardness) was selected. The 35HGS steel parts are characterized by very high strength properties, considerable ductility, elongation after quenching (at 860–880 °C), and high-temperature tempering (at 500–650 °C). This steel grade is used for heavily loaded components of medium-size machinery, such as shafts, connecting rods, spindles, axles for trucks and tractors, and levers. The 35HGS steel is delivered as die forgings, forgings, rods, and hot-rolled sheets.

Table 3 gives the chemical composition of the workpiece (35HGS alloy steel for tempering), while the mechanical properties of this steel grade are given in Table 4.

It should be emphasized that 35HGS steel is characterized not only by very high tensile strength after quenching and tempering, but also by high abrasion resistance. Hence, this steel grade is widely used for the manufacture of critical machine components.

## 3. Results

### 3.1. Coating-Substrate Adhesion

Values of the critical load *L_c_* obtained for the three different types of hard coatings applied to the sintered carbide cutting inserts are listed in Table 5.

**Figure 3:**
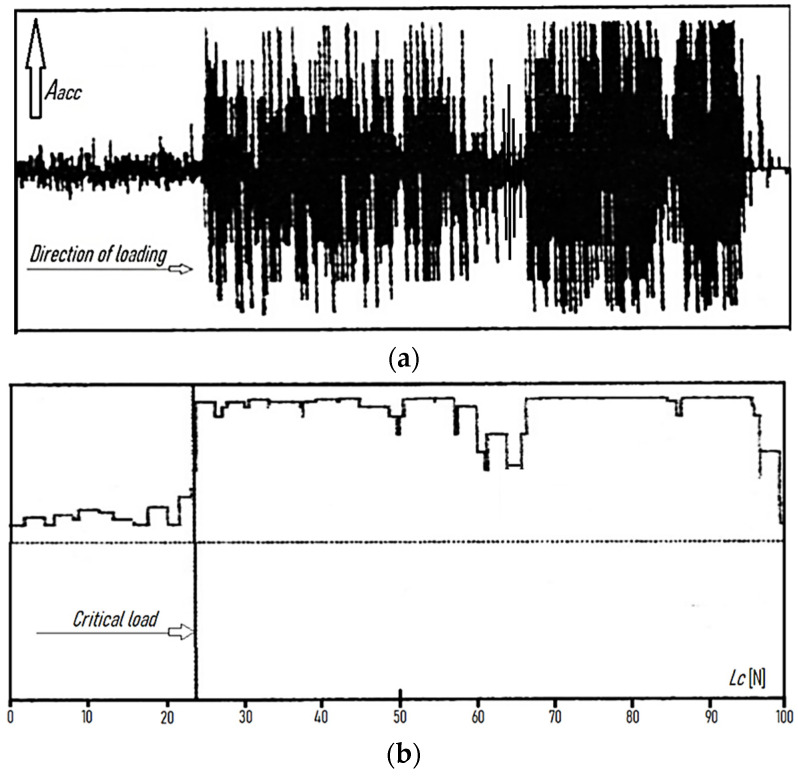

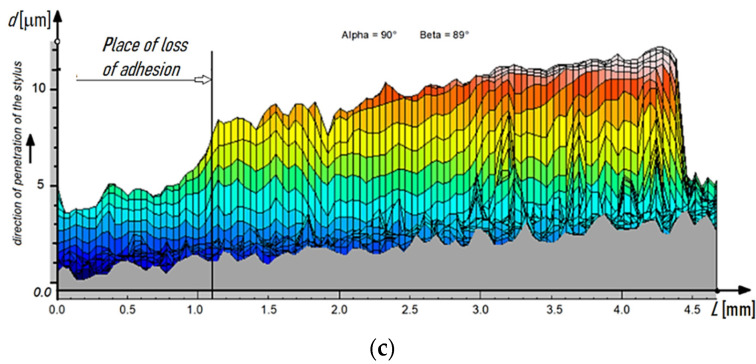
Examples of scratch test results obtained for the TiN coating applied to the cemented carbide cutting inserts SM25 (P15–P40, M25–M35) before laser heating: (**a**) actual value of the vibration signal (vibration acceleration signal amplitude—*A_acc_*), (**b**) positive envelope of the vibration signal as a function of loading, and (**c**) negative image of the scratch occurring with a gradual increase in the load on the indenter.

**Figure 4:**
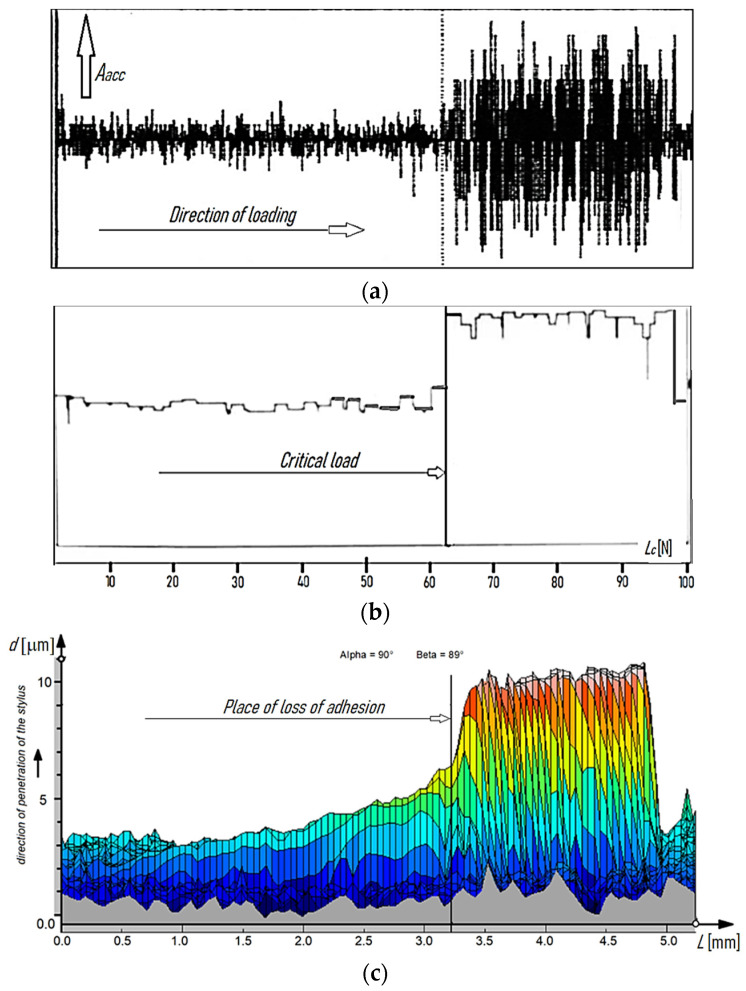
Examples of scratch test results obtained for the TiN coating applied to the cemented carbide cutting inserts SM25 (P15–P40, M25-M35) after laser heating: (**a**) actual value of the vibration signal (vibration acceleration signal amplitude—*A_acc_*), (**b**) positive envelope of the vibration signal as a function of loading, and (**c**) negative image of the scratch occurring with a gradual increase in the load on the indenter.

The 2D images were recorded as positives and negatives. The 2D negative images proved useful in estimating the critical load *L_c_*, while the positive images (primarily 3D) were used to compare the cutting tool surface topographies before and after laser heating. Figure 3 and Figure 4 show exemplary printouts of the scratch test obtained for the TiN coating applied to the cemented carbide cutting inserts before and after laser heating with 40% of the maximum laser power, considering the actual vibration signal value (vibration acceleration signal amplitude—*A_acc_*), the positive envelope of vibration signal as a function of loading, and the negative image of the scratch of the TiN coating on the SM25 substrate that occurred with a gradual increase in the load on the diamond indenter.

Figure 3a,b and Figure 4a,b demonstrate a significant difference between the values of the adhesion strength of the TiN coating to the SM25 cemented carbide substrate before and after laser heating with 40% of the maximum laser power. Figure 3c and Figure 4c show examples of how 2D images from the profilometer can be used to illustrate the positive effect of laser heating on the TiN coating adhesion. As previously mentioned, the load was gradually increased in the scratch test (from left to right, as shown in Figure 3 and Figure 4). The sudden increase in the thickness of the protrusion (Figure 3c and Figure 4c) and in the values of the vibration signal (Figure 3a,b and Figure 4a,b) indicates a loss of the coating adhesion to the substrate. As can be seen from Figure 3 and Figure 4, a nearly threefold higher load was required to detach the coating from the substrate after laser heating (63 N/23 N).

**Figure 5:**
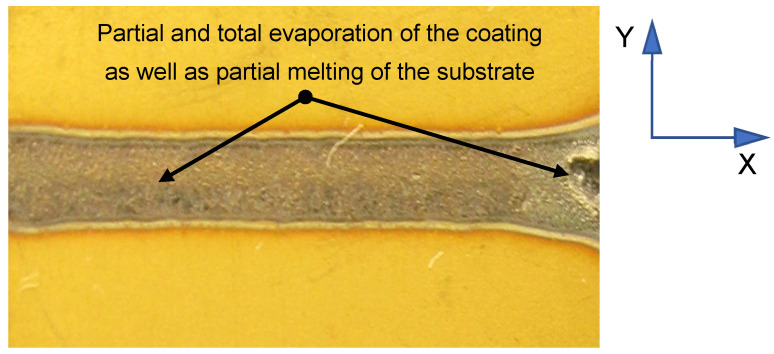
Partial and total evaporation of the coating, as well as partial melting of the substrate, observed after laser heating conducted with a laser beam power density of 12,420 W/cm^2^ (60% of the maximum laser power).

The 3D positive images of the laser-heated cutting tool surface obtained with a profilometer are not shown here due to the fact that SEM images of the tool surface are shown later in this paper. Secondary electron images (SEIs) clearly demonstrate that the surface morphology changed with increasing the laser beam power. In laser heating conducted with 60% of the maximum laser power, one can observe either total or partial evaporation of the coating, as well as partial melting of the substrate (Figure 5).

**Figure 6:**
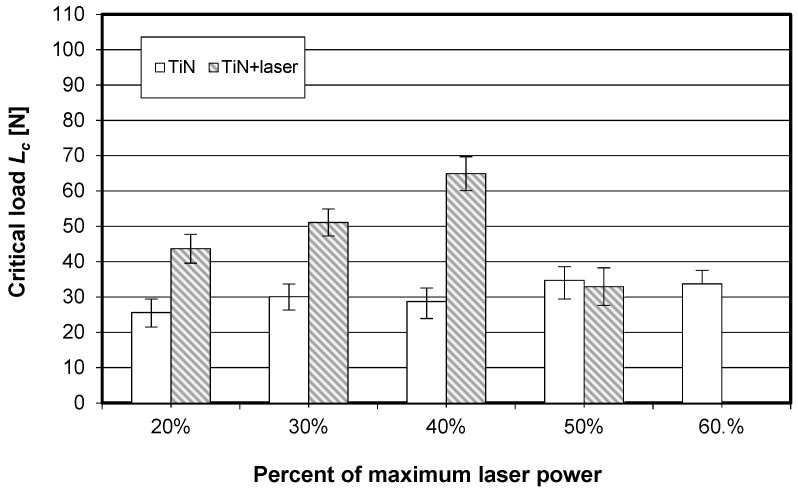
Impact of laser heating on the adhesion (critical load determined by scratch test) of the single-layer coating (TiN) to the SM25 sintered carbide cutting inserts (P15–P40).

**Figure 7:**
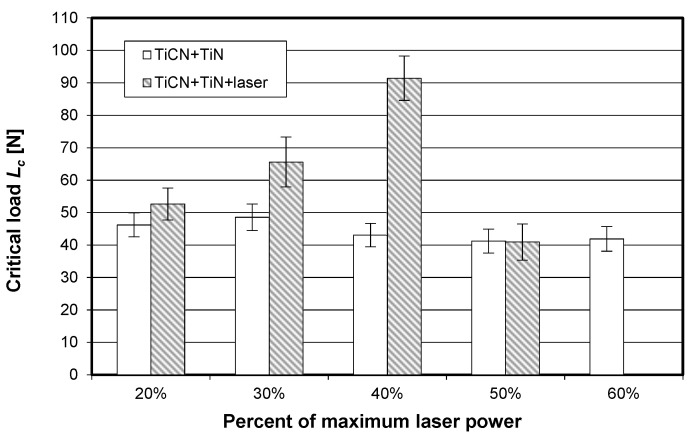
Impact of laser heating on the adhesion (critical load determined by scratch test) of the double-layer coating (TiCN + TiN) to the SM25 sintered carbide cutting inserts P15–P40).

**Figure 8:**
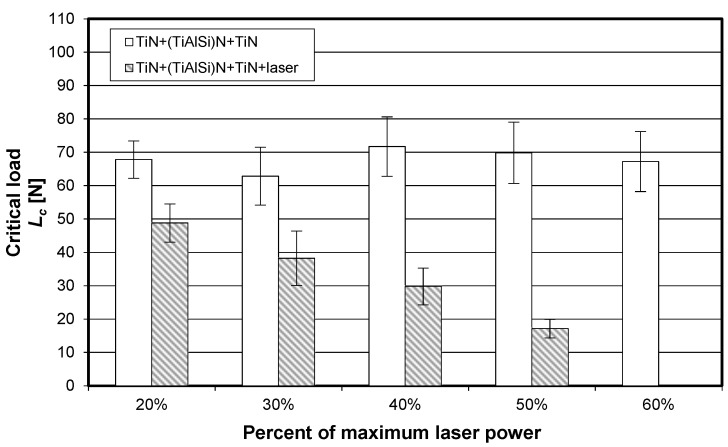
Impact of laser heating on the adhesion (critical load determined by scratch test) of the triple-layer coating (TiN + (TiAlSi)N + TiN) to the SM25 sintered carbide cutting inserts (P15–P40).

The visually observed absence of the TiN coating after laser heating conducted with 60% of the maximum laser power was also confirmed by X-ray fluorescence spectrometry. Figure 6, Figure 7 and Figure 8 show the effect of laser heating on the adhesion of single-, double-, and triple-layer coatings to the sintered carbide cutting inserts.

It can be seen from Figure 6 that laser heating significantly improved the TiN coating adhesion only when the laser beam power density ranged between 20% and 40%. It can be observed that the confidence intervals marked in Figure 6 did not overlap, and that the mean critical load values were higher after laser heating than before it.

Figure 7 reveals that, for the TiCN + TiN coating, the mean critical load significantly increased when laser heating was conducted with 30% and, particularly, 40% of the maximum laser power. A different trend can be observed for the triple-layer coating (TiN + (TiAlSi)N + TiN), which is shown in Figure 8. For this case, a significant decrease in *L_c_* can be observed for all applied laser power values. It can be assumed that the presence of the silicon–aluminum nitride layer with low thermal shock resistance in rapid heating had a negative effect on the adhesion of this triple-layer coating.

The difference between the thermal expansion coefficients of the silicon nitride coating (~2.9 × 10^−6^ K^−1^) and the SM25 sintered carbide substrate (~8.0 × 10^−6^ K^−1^) is significant [45]. In contrast, the difference in the thermal expansion coefficients of SM25 sintered carbide and TiCN (~8.0 × 10^−6^ K^−1^) and TiN (~9.35 × 10^−6^ K^−1^) coatings is very small [45]. That means that the TiN and TiNC + TiN coatings exhibit higher resistance to laser heating.

**Figure 9:**
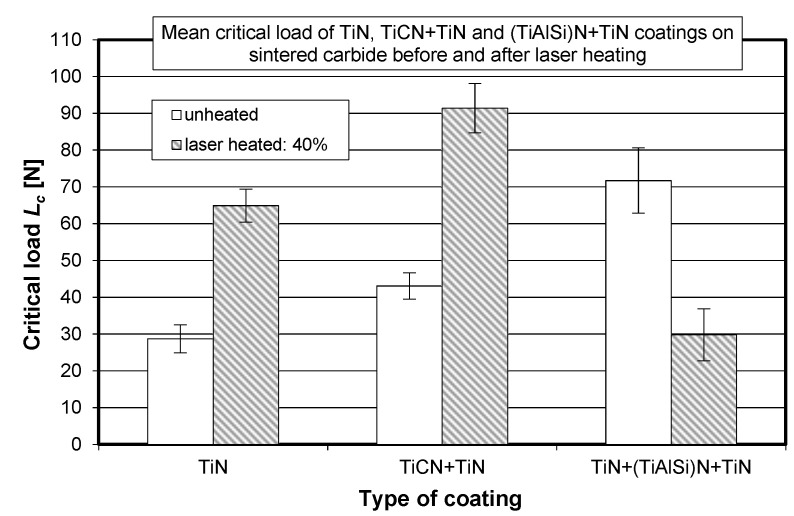
Effect of laser heating (with 40% of the maximum laser power) on the adhesion of single-layer (TiN), double-layer (TiCN + TiN), and triple-layer (TiN + (TiAlSi)N) + TiN) coatings to the replaceable cutting insert made of SM25 sintered carbide.

Furthermore, another significant difference between the silicon–aluminum nitride coatings and the TiCN and TiN coatings depends on the type of chemical bonds that prevail in these compounds. Titanium carbide (TiCN) and titanium nitride (TiN) have covalent and metallic bonds, but silicon–aluminum nitride has predominantly ionic bonds. Materials with predominantly ionic bonding such as Si_3_N_4_, SiO_2_, Al_2_O_3_, BeO, and ZrO_2_ are brittle chemical compounds [6,13,46]. This means that extensive cracking will occur if mechanical load exceeds the elastic limit. Moreover, rapid laser heating causes thermal shock in these compounds. Figure 9 shows a comparison of the adhesion of single-, double-, and triple-layer coatings to the sintered carbide substrate, obtained for the most favorable laser heating case (40% of the maximum laser power).

**Figure 10:**
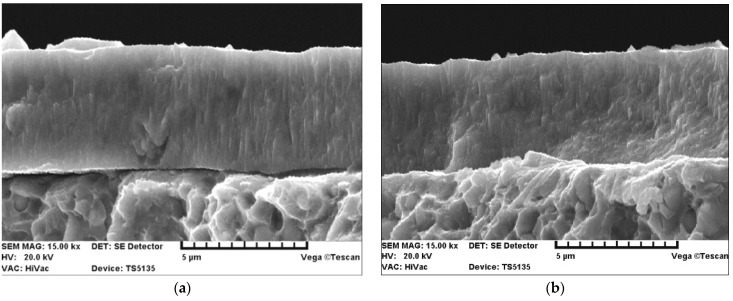
SE images of the fracture of the TiN coating (**a**) before and (**b**) after laser heating with 40% of the maximum laser power.

For a more detailed explanation of the adhesion test results, the SE images of the fracture of the TiN coating applied to the replaceable cutting insert are additionally presented in Figure 10 and then discussed. Figure 10 shows a comparison of the SE images of the fracture of the TiN coating before and after laser heating using 40% of the maximum laser power.

The SE images show that there was improved adhesion (closer contact) of the TiN coating to the substrate after laser heating. Similar observations were made for several other fractures.

### 3.2. Cutting Tool Wear and Life in a Turning Process

**Figure 11:**
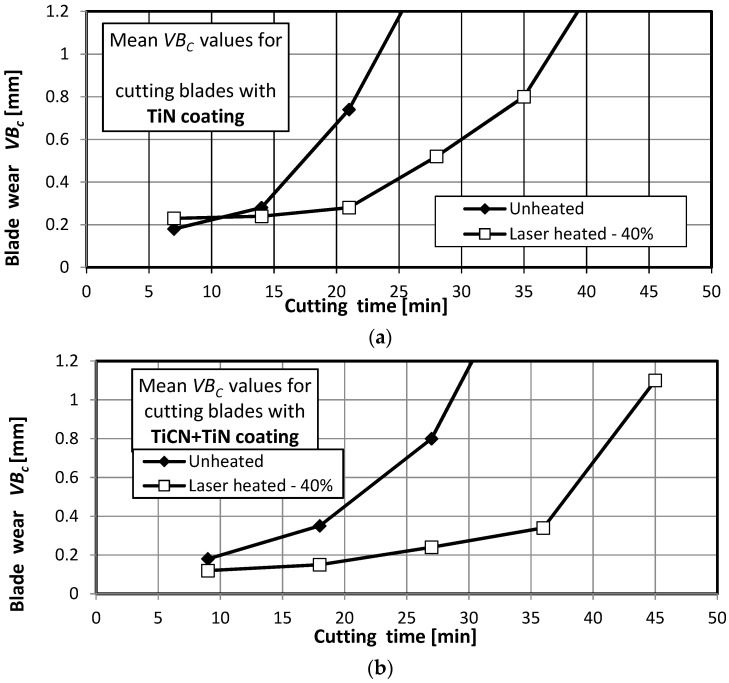

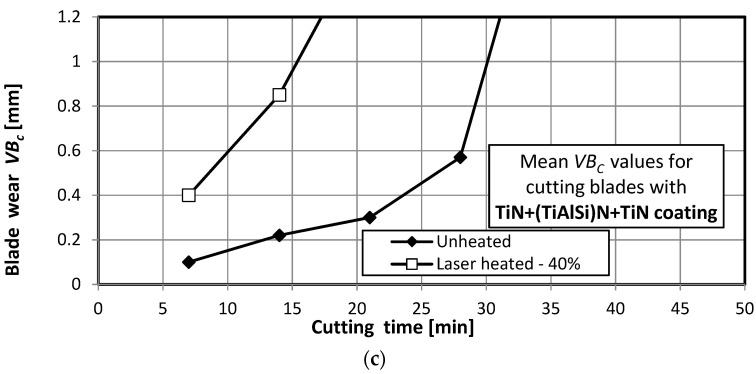
Examples of wear curves for the unheated and laser-heated (40% of the maximum laser power) SM25 sintered carbides cutting blades coated with (**a**) TiN, (**b**) TiCN + TiN, and (**c**) TiN + (TiAlSi)N + TiN, obtained in a turning process of 35HGS steel.

The wear and life of the cutting blades were tested in accordance with the conditions specified in Section 2. Figure 11 shows wear curves for the unheated and laser-heated (with 40% of the maximum laser power) SM25 sintered carbide cutting blades coated with TiN, TiCN + TiN, and TiN + (TiAlSi)N + TiN, obtained in a turning process of 35HGS steel.

**Figure 12:**
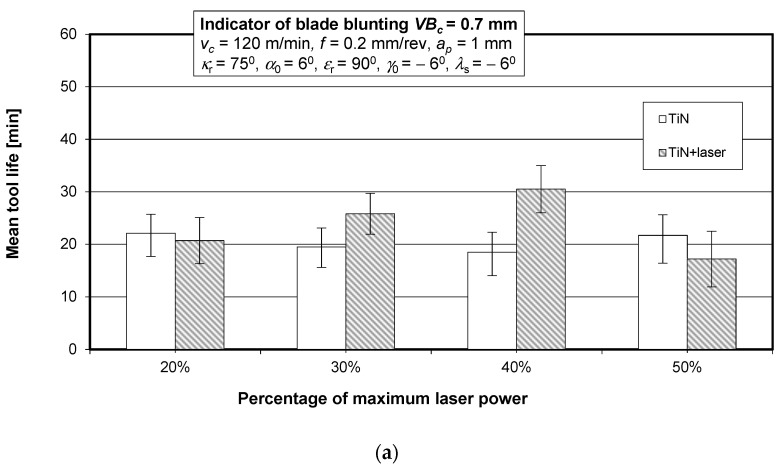

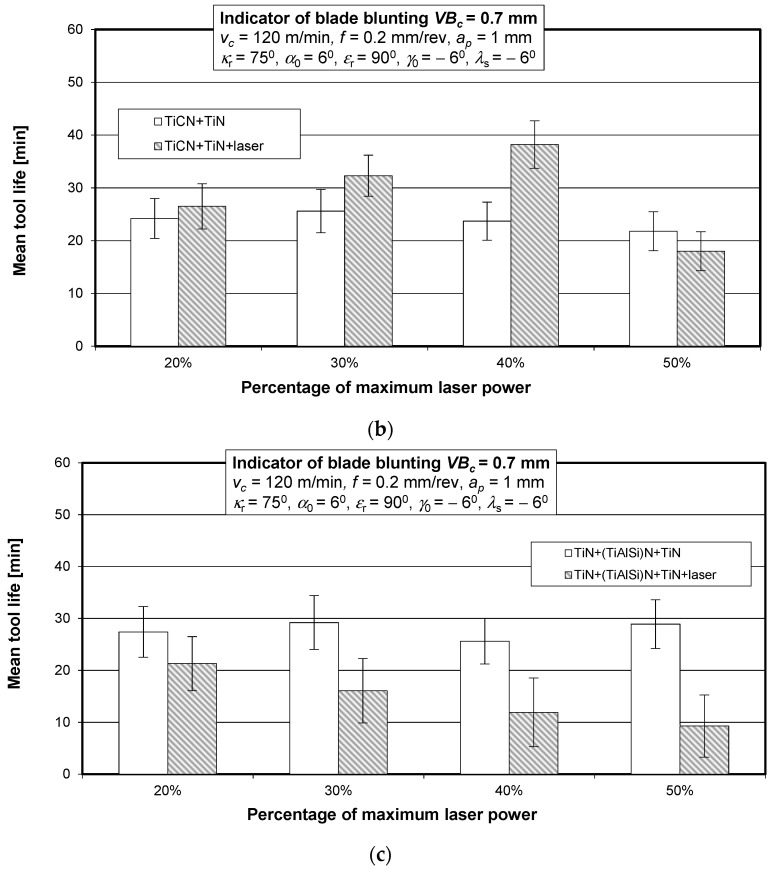
Mean life of the SM25 sintered carbide cutting blades coated with (**a**) TiN, (**b**) TiCN + TiN, and (**c**) TiN + (TiAlSi)N + TiN before and after laser heating with different laser power values, obtained in a turning process of 35HGS alloy steel.

The wear curves were used to determine the cutting tool life for a blade blunting indicator of *VB_c_* = 0.7 mm. Figure 12 shows the mean life of the SM25 sintered carbide cutting tool blades coated with TiN, TiCN + TiN, and TiN + (TiAlSi)N + TiN before and after laser heating with different laser power values, obtained in a turning process of 35HGS steel.

**Figure 13:**
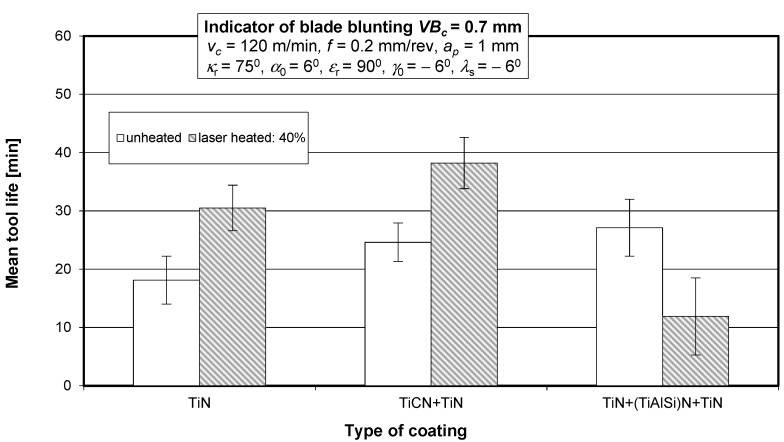
Mean life of the SM25 sintered carbide cutting tool blades coated with TiN, TiCN + TiN, and TiN + (TiAlSi)N + TiN before and after laser heating with 40% of the maximum laser power, obtained in a turning process of 35HGS alloy steel.

Figure 13 shows the mean life of the SM25 sintered carbide cutting tool blades coated with TiN, TiCN + TiN, and TiN + (TiAlSi)N + TiN before and after laser heating conducted with 40% of the maximum laser power, obtained in a turning process of 35HGS alloy steel.

**Figure 14:**
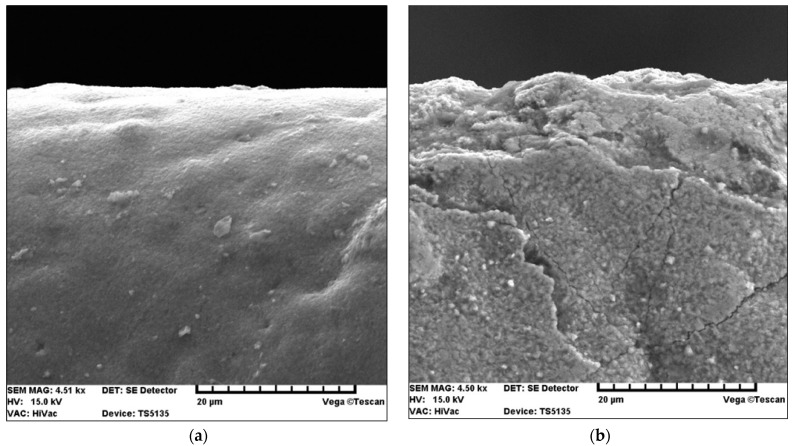
Secondary electron images (SEIs) of SM 25 cutting tool blades coated with (**a**) TiN and (**b**) TiN + (TiAlSi)N + TiN, after heating with 40% of the maximum laser power.

Figure 14 shows secondary electron images (SEIs) of the cutting tool blades coated with TiN and TiN + (TiAlSi)N + TiN after laser heating conducted with 40% of the maximum laser power.

The above SE images confirm that the large difference between the thermal expansion coefficients of the coating (TiAlSi) N and the SM25 cemented carbide substrate had a negative effect [45], because it led to the formation of cracks in this coating during laser heating. On the other hand—as previously mentioned—the difference between the thermal expansion coefficients of SM25 cemented carbides and TiCN and TiN coatings is very small [45]. Hence, the TiN coating exhibits a higher resistance to laser heating. Another cause of the cracking of the coating with a (TiAlSi) N layer as a result of laser heating is the predominance of ionic bonds in this compound occurring in brittle materials. The TiN coating, similarly to the TiCN coating, has covalent and metallic bonds.

**Figure 15:**
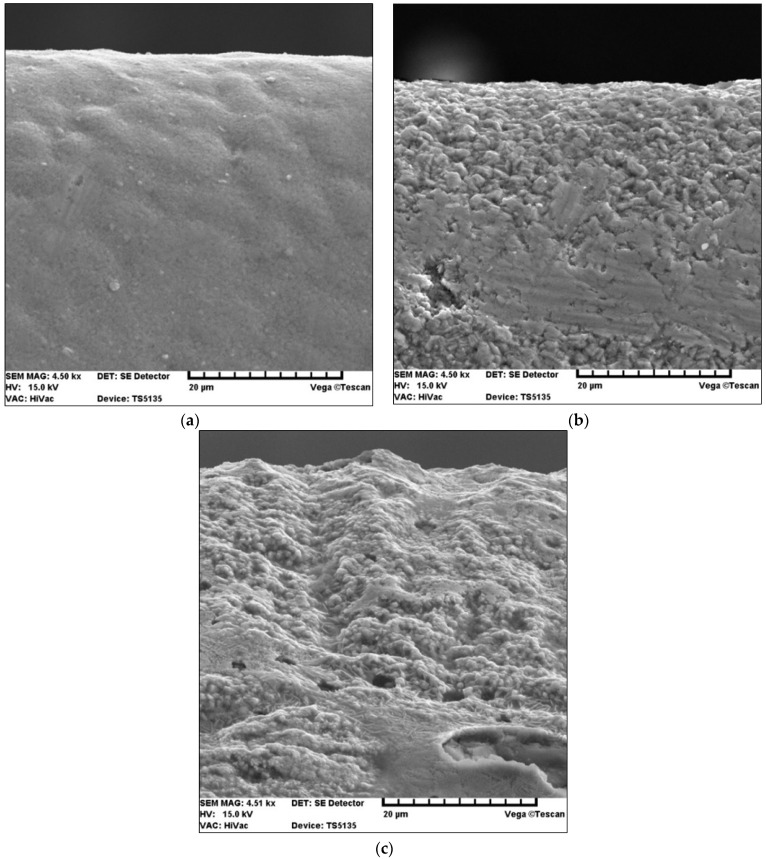
Secondary electron images (SEIs) of SM25 cutting blades coated with TiCN + TiN, after heating with (**a**) 40%, (**b**) 50%, and (**c**) 60% laser power.

Figure 15 shows secondary electron images (SEIs) of the SM25 sintered carbide cutting tool blades coated with TiCN + TiN after laser heating conducted with 40%, 50%, and 60% of the maximum laser power.

**Figure 16:**
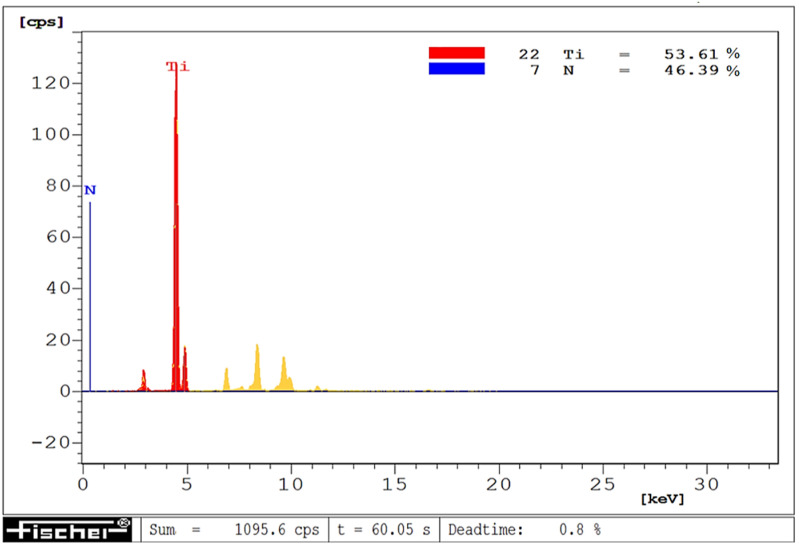
EDXRF image of a TiN coating before laser heating.

**Figure 17:**
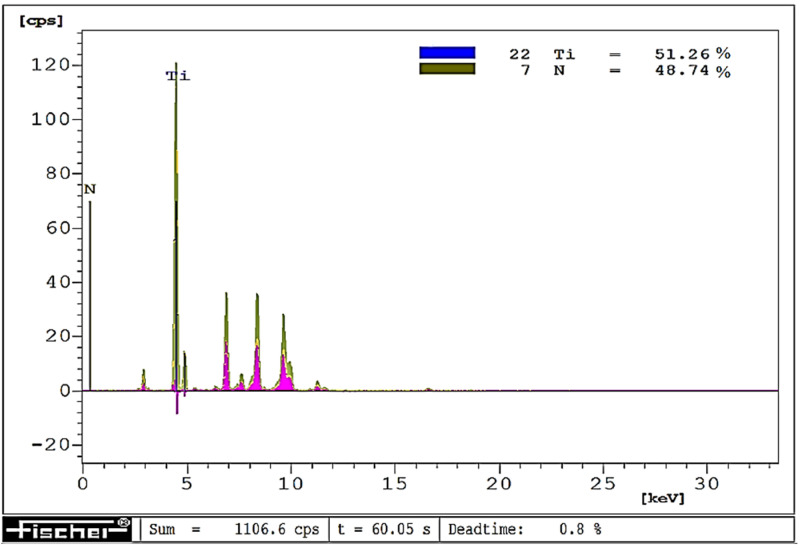
EDXRF image of a TiN coating after laser heating with 40% of maximum laser power.

**Figure 18:**
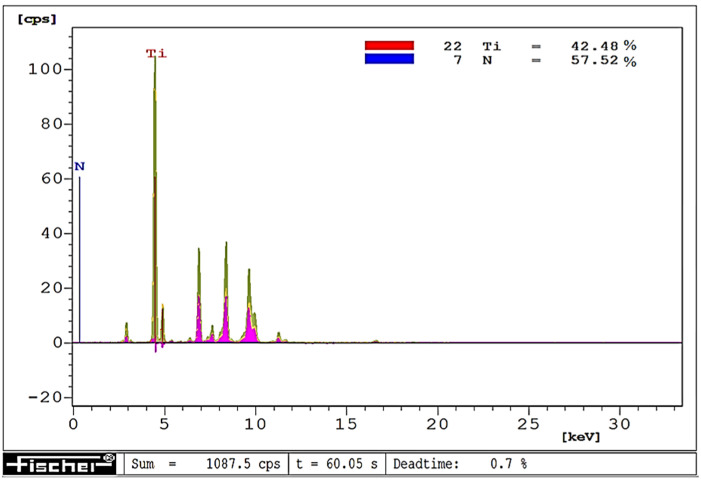
EDXRF image of a TiN coating after heating with 50% of maximum laser power.

The coating adhesion to the substrate, as well as the cutting tool life, can be affected by changes in the coating material chemical composition induced by laser heating. To give an example, prior to laser heating, the tested titanium nitride coatings contained on average 55% titanium and 45% nitrogen, whereas, after laser heating conducted with 40% of the maximum laser power, their chemical composition was almost stoichiometric (on average 50.5% titanium and 49.5% nitrogen). On the other hand, after laser heating with 50% of the maximum laser power, the coating contained only 42% titanium and as much as 58% nitrogen. Examples of chemical microanalysis images are given in Figure 16, Figure 17 and Figure 18.

**Figure 19:**
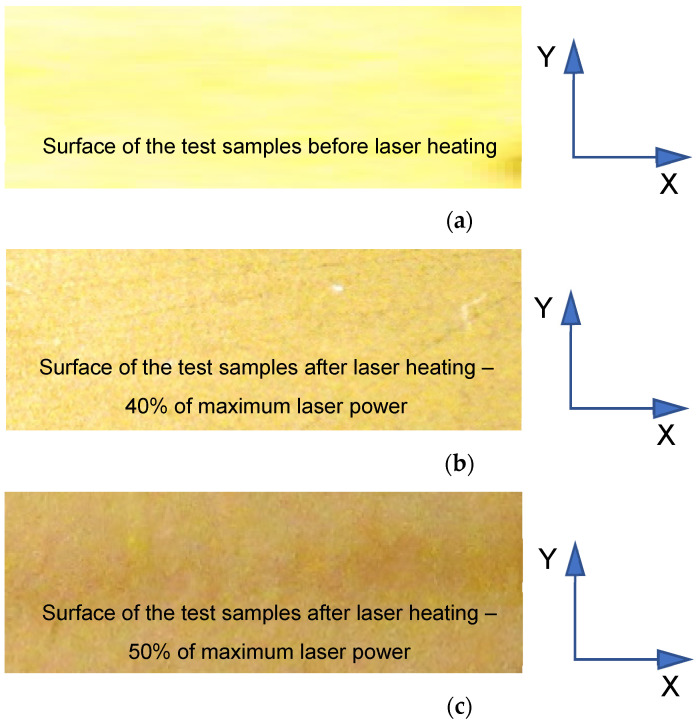
Images showing the surface of the samples before (**a**) and after laser heating with (**b**) 40% and (**c**) 50% of maximum laser power.

Before laser heating, the TiN coatings were light yellow. After heating with 40% of the maximum laser power, their color changed to golden, whereas, after heating with 50% of the maximum laser power, the color became darker (golden brown). The color changes observed for the TiN coatings were confirmed by chemical microanalysis results [6,46,47,48,49]. Figure 19 shows images of the surface of the test samples before (a) and after laser heating with (b) 40% and (c) 50% of the maximum laser power.

The chemical composition of the TiN coating has a significant influence on its mechanical properties. As for the group IVb transition metal nitrides, i.e., TiN, ZrN, and HfN (as in the case of carbides of the same transition metals), they reach the highest hardness for the stoichiometric composition, i.e., for N/Me = 1 (C/Me = 1) and, therefore, the highest abrasive wear resistance [49,50]. A greater proportion of transition metal in the coating results in a softer and less abrasive wear-resistant coating. On the other hand, the excess of nitrogen in the TiN coating in relation to the stoichiometric composition causes a rapid increase in the brittleness of the coating [50,51]. This was confirmed, inter alia, by our previous study [6]. As shown by data reported in the literature [15], the diffusion penetration of the coating components to a depth of only 1 µm is observed due to a very short laser heating time. Increasing the diffusion is only possible with simultaneous remelting of the coating material and the surface layer of the substrate by laser surface alloying (LSA); however, this applies to a completely different coating technology, which is not applicable to cutting tools [15]. Laser heating can also significantly help fill gaps in the structure of the transition layer between the coating and the substrate. This process leads to improved mechanical properties of the transition layer and increased mechanical anchoring of the coating to the substrate, thereby improving its adhesion. The SE images show improved adhesion (closer contact) of the TiN coating to the substrate after laser heating the replaceable cutting insert. Similar observations were made for several other fractures.

As mentioned in Section 1, previous studies [35,36] by the coauthor of this paper focused on coatings produced using the high-temperature CVD process. In those studies, two variants of heating by laser beam were employed:

- One-step heating by a laser beam with different power densities,
- Two-step combined laser heating with different power densities, comprising (1) preliminary heating and (2) principal heating.

The results showed that, among other things, the use of two-stage heating with different power density values did not have a positive effect on the coating adhesion to the substrate because it led to an unfavorable change in the chemical composition of the coatings. There was an advantage of the nitrogen content in the TiN coating and of carbon in the TiC coating over the titanium content, which led to an undesirable increase in the brittleness of these coatings. For this reason, in this study, two-stage heating was not used.

## 4. Conclusions

This study demonstrated that laser heating is a useful means of improving the adhesion of hard coatings to cemented carbides cutting tools and increasing the tool life in a turning process. In particular, the following conclusions were drawn:

(a) There exists a direct correlation between the adhesion of the tested hard coatings to the surface of sintered carbide cutting tool blades and the life of these replaceable cutting inserts in a turning process of 35HGS steel;
(b) Laser heating increases the adhesion of PVD coatings to the substrate, but the degree of this increase depends on the laser power and hard coating type;
(c) The results showed that *L_c_* and tool life can be significantly increased by using a laser beam power density not exceeding 8280 W/cm^2^ but only for single-layer and double-layer coatings. The use of a higher laser beam power (50% of the maximum laser power) led to reduced coating–substrate adhesion and shorter cutting tool life in a turning process of quenched and tempered 35HGS alloy steel specimens;
(d) The use of a higher laser beam power (60% of the maximum laser power) led to complete evaporation of the coating and partial melting of the substrate;
(e) A silicon–aluminum nitride coating layer is not recommended for high-speed laser heating due to its low resistance to thermal shock and high risk of cracking under these conditions. In addition, silicon and aluminum nitrides with ionic chemical bonding are more brittle chemical compounds than TiN and TiCN.

## Figures and Tables

**Table 1:** Mechanical properties and chemical analysis of the SM25 (P15–P40, M25–M35) cemented carbides (according to PN-81/H-89500).

SM25: P15–P40, M25–M35			
Mechanical Properties		Chemical Analysis	
Hardness:	15.5 GPa	WC:	69.5%
Bending strength:	2000 MPa	Co:	9.5%
Density:	12.6 g/cm^3^	TiC + TaC–NbC:	21.0%

**Table 2:** Laser heating parameters employed in experiments.

Parameters of Laser Heating			
% of max. Laser Power	Power Density (W/cm^2^)	Diameter of Laser Beam (mm)	Scanning Speed (m/min)
20	4140		
30	6210		
40	8280	4.0	1.3
50	10,350		
60	12,420		

**Table 3:** Chemical composition of 35HGS steel (according to PN-72/H-84030).

C	Mn	Si	Cr
0.32 ÷ 0.40	0.80 ÷ 1.10	1.10 ÷ 1.40	1.10 ÷ 1.40
**Ni**	**Mo**	**V**	**W**
0.30	0.10	0.05	0.20
**Ti**	**Cu**	**P**	**S**
0.05	0.25	≤0.035	≤0.035

**Table 4:** Mechanical properties of 35HGS steel (according to PN-72/H-84030).

Quantity	Mark	After Quenching and Tempering
Tensile strength	*R_m_*	≥1620 MPa
Yield point	*R_e_* (*R_p0,2_*)	≥1280 MPa
Elongation	*A*	≥9%
Hardness	*HRC*	max 32
Necking	*Z*	≥40%

**Table 5:** Adhesion of the single-, double-, and triple-layer coatings to the sintered carbide substrates before and after laser heating conducted with different laser powers.

Laser Heating Conditions (% of Max. Laser Power)	Type of Coating		
	TiN	TiCN + TiN	TiN + (TiAlSi)N + TiN
	Critical Load Values *L_c_* (N)		
0	25.60 ± 3.8	47.32 ± 3.7	67.53 ± 5.6
20	43.67 ± 4.0	52.66 ± 4.9	48.79 ± 5.7
30	51.09 ± 3.8	65.61 ± 7.7	38.23 ± 8.2
40	**64.89 ± 5.8**	**91.42 ± 6.8**	29.79 ± 5.5
50	32.95 ± 3.9	40.93 ± 5.6	17.12 ± 2.8
60	Total evaporation of coating and partial melting of substrate		

## Data Availability

Not applicable.
